# Photon-counting CT: image quality evaluation in patients with tibial plateau fracture treated with metallic osteosynthesis material

**DOI:** 10.1186/s41747-026-00761-8

**Published:** 2026-06-19

**Authors:** Ann-Sofi Björkman, Alexandr Malusek, Anders Persson, Håkan Gauffin, Seppo K. Koskinen

**Affiliations:** 1 Department of Health, Medicine and Caring Sciences, Linköping University, Linköping, Sweden; 2 Clinical Department of Radiology in Linköping, Region Östergötland, Linköping, Sweden; 3 Center for Medical Image Science and Visualization (CMIV), Linköping University, Linköping, Sweden; 4 Department of Biomedical and Clinical Sciences, Linköping University, Linköping, Sweden; 5 Clinical Department of Orthopedics in Linköping, Region Östergötland, Linköping, Sweden; 6 Department of Clinical Science, Intervention and Technology, Division of Radiology, Karolinska Institutet, Stockholm, Sweden

**Keywords:** Artifacts, Fracture fixation (internal), Fractures, Knee joint, Tomography (X-ray computed)

## Abstract

**Objective:**

The aim was to compare the image quality of photon-counting detector CT (PCD-CT) and energy-integrating detector CT (EID-CT) in patients with tibial plateau fractures treated with metallic osteosynthesis material, and to identify optimal reconstruction parameters for PCD-CT.

**Materials and methods:**

After ethical approval, twelve patients underwent PCD-CT and EID-CT scans. Images were reconstructed using bone and soft-tissue kernels with metal artifact reduction (iMAR). PCD-CT virtual monoenergetic images (VMI) at 70, 110, and 150 keV were generated. Five radiologists assessed metal artifact severity and bone and soft-tissue visualization using a 7-point Likert scale. Visual grading characteristics analysis was performed. Noise levels were quantified and compared using Wilcoxon’s signed-rank test.

**Results:**

EID-CT was rated superior in reducing metal-artifact streaks (AUC: 0.10–0.21). No significant difference was found between EID-CT iMAR and PCD-CT VMI at 110 keV and 150 keV (AUC: 0.40–0.49) concerning the metal-bone interface. An ultra-high-resolution PCD-CT kernel outperformed its EID-CT counterpart in bone visualization, with AUC values of 0.67–0.92 across all bone criteria, including those incorporating artifact-affected regions. PCD-CT showed lower noise.

**Conclusion:**

Metal artifact reduction was superior in EID-CT iMAR images compared to PCD-CT iMAR and VMI 110/150 keV, except at the metal-bone interface, where EID-CT iMAR images and PCD-CT VMI 110/150 keV performed comparably. The ultra-high-resolution PCD-CT kernel provided the best bone visualization, even when artifact-affected areas were included. Noise levels were lower in PCD-CT. This is the first *in vivo* comparison of photon-counting and energy-integrating CT for postoperative knee imaging with metallic osteosynthesis material.

**Relevance statement:**

These findings highlight the need for improved metal artifact reduction in PCD‑CT, while demonstrating superior bone visualization, and support a complementary interpretation strategy in postoperative knees using high‑energy VMI and MAR‑corrected reconstructions.

**Key points:**

- Photon-counting CT demonstrated excellent bone visualization.
- Conventional EID-CT achieved better metal artifact reduction than photon-counting CT.
- The metal-bone interface was rated similarly for energy-integrating CT and photon-counting CT virtual monoenergetic images.
- These findings indicate that further improvements in metal artifact reduction algorithms for PCD-CT are warranted.

**Graphical Abstract:**

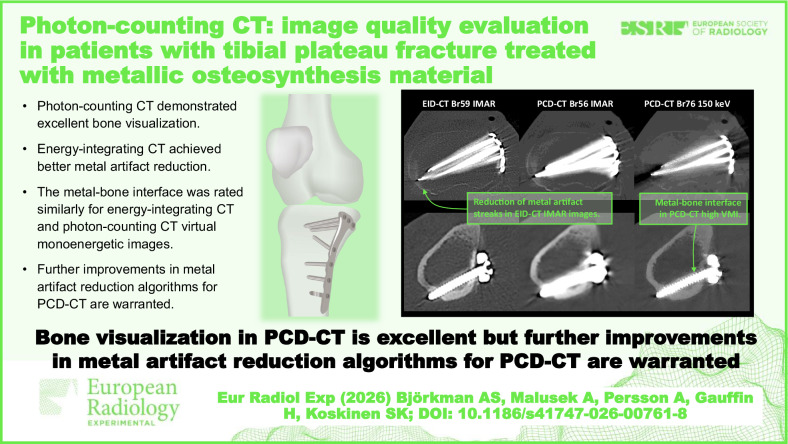

## Background

In musculoskeletal (MSK) imaging, good visualization of bone and soft tissues is important. Metal artifacts from orthopedic implants are a common problem, as the artifacts can obscure anatomical structures, as well as complications such as implant loosening and fluid accumulation. Metal artifacts in CT often present as bright and dark streaks radiating from metal objects or blooming, where the metal implant looks larger than it really is, obscuring the tissue in the close vicinity [1]. Sometimes, metal artifacts are difficult to see, appearing as thin radiating streaks of almost the same HU value as the tissue itself [1, 2]. The main causes of metal artifacts are photon starvation and beam hardening [1, 3].

Several metal artifact reduction (MAR) techniques are available. Firstly, scanner parameters can be adjusted, increasing the x-ray tube current, as well as the energy of photons, but this will come at the cost of a higher radiation dose. Secondly, all commercially available CT scanners provide post-processing methods, MAR algorithms, commonly by replacing missing data with interpolation [1, 3]. MAR algorithms reduce artifacts but come with some drawbacks: Reconstruction kernel sharpness is often restricted, which can hinder detailed bone visualization. Bone visualization adjacent to metal implants can be impaired [4–7], and MAR processing can introduce new artifacts or even cause metallic implants to disappear [1, 8–10].

Virtual monoenergetic images (VMI), typically at high energies of 100–150 keV, have also, either alone or in combination with MAR algorithms, been suggested as a problem-solver for metal artifacts [6, 11, 12]. Such images can be created with dual-energy CT (DECT) or, more recently, with photon-counting detector CT (PCD-CT). The PCD-CT uses a new type of detector that differs from the conventional energy-integrating detector (EID) in that the energy of individual photons is recorded, giving inherent spectral properties [13]. In a previous study, a bovine knee with an implanted metal plate and screws was used to compare image quality between DECT and a PCD-CT prototype. High VMI (150 keV) acquired with the PCD-CT, without a MAR algorithm, due to the unavailability of such algorithms for that system, were rated as comparable to or superior to DECT images reconstructed with a MAR algorithm [14]. The development of this PCD-CT prototype subsequently led to the first commercially available whole-body PCD-CT.

MAR on that PCD-CT has been investigated using various approaches, primarily through comparisons of different VMI levels, either alone or in combination with a MAR algorithm. While VMI alone may be sufficient for reducing metal artifacts from spinal fixation, findings for hip arthroplasty are more divergent, with some studies reporting satisfactory results using VMI alone and others favoring the use of MAR algorithms [15–20]. Moreover, high-energy VMI has been associated with potential drawbacks, including reduced image contrast [20] and overcorrection of artifacts [17]. Only a limited number of studies have directly compared PCD-CT with EID-CT in the context of metal artifacts, reporting no significant difference between systems in patients with spinal instrumentation and in a single cadaveric wrist with a plate [21, 22].

In addition to artifact reduction, accurate visualization of bone and surrounding soft tissues is essential, requiring high spatial resolution and adequate contrast-to-noise ratio. The PCD-CT inherently provides higher resolution than EID-CT because it does not require septa between detector elements. PCD-CT also reduces electronic noise by applying a low-energy threshold that removes signals below a predefined level, which is expected to further improve image quality [13, 23]. In tibial plateau fractures, fractures involving the weight-bearing joint surface, even small step-offs exceeding 2 mm, have been associated with adverse outcomes such as osteoarthritis [24], highlighting the need for both high-resolution imaging and effective artifact reduction in the postoperative setting. Prior PCD-CT studies demonstrate excellent depiction of fine bony details using very sharp kernels [15, 25–30], but detailed postoperative knee evaluation remains insufficiently studied.

The aim of the study was to compare a whole-body clinical PCD-CT with a state-of-the-art EID-CT for MAR and visualization of bone and soft tissue, as well as to find suitable reconstruction settings for the PCD-CT for imaging of the post-surgical knee.

## Material and methods

### Patients

Ethical approval was obtained from the regional review board (2021–02987). Patients were recruited from the orthopedic ward at Linköping University Hospital. Eligible patients were those with a fracture of the tibial plateau requiring surgical fixation and being able to provide informed consent. All participants gave informed consent.

Twelve consecutive patients were recruited between November 2022 and October 2023. Mean age was 46 years (range 20–77), and median BMI was 26 (range 21–39). The cohort comprised six men and six women. Injuries resulted from falls in the same plane, bicycle or electric scooter accidents, motorcycle accidents, horseback riding, football, and skiing. Nine were injured in the left knee, and three in the right. Fractures were classified by Schatzker’s method: Type 2 (*n* = 4), Type 3 (*n* = 1), Type 4 (*n* = 1), Type 5 (*n* = 3), and Type 6 (*n* = 3). Median time from injury to surgery was 5.5 days (range 2–12). Osteosynthesis used stainless steel implants, consisting of one plate in eight cases, two plates in three cases, and screws only in one case. One patient had a contralateral knee prosthesis. Median time from surgery to imaging was 1 day (range 1–15). All patients were examined in both scanners on the same day.

### Imaging

#### Scanner protocol

Patients were scanned in an EID-CT (Somatom Force, Siemens Healthcare) using the clinical protocol (Sn150 kV, quality reference mAs 300 mGy, rotation time 1 s, pitch 0.6, collimation 192/0.6 mm), and in a PCD-CT (Naeotom Alpha, version VA50 and VA50SP1, Siemens Healthcare) using an ultra-high resolution (UHR) 140 kV protocol (pitch 0.85, rotation time 1 s, collimation 120/0.2 mm). The CTDIvol selected by the EID-CT was recorded and matched on the PCD-CT. Median CTDI was 3.4 mGy (95% CI: 2.9-4.4) for EID-CT and 3.2 mGy (95% CI: 2.5–4.2) for PCD-CT. In three cases, the PCD-CT scan was inadvertently performed at a lower dose than the EID-CT scan (2.15 *versus* 3.65 mGy; 1.78 *versus* 2.63 mGy; 2.01 *versus* 3.7 mGy). Although this could potentially bias the PCD‑CT assessments, visual review showed no apparent impact on image quality, noise levels were comparable, and PCD‑CT images showed high scores across several evaluation criteria. These deviations were therefore considered minor, and the cases were retained in the analysis. The median image quality (IQ) value for the entire cohort was 26.5 (range 15–46), compared to 28 (range 22–46) for patients with matched CTDI_vol_. Scanning was performed supine with both knees in the scan field. Positioning was replicated as closely as possible for the subsequent PCD-CT scan.

#### Post-processing

To minimize the risk of readers identifying the scanner type by the slice thickness or matrix size, the minimal slice thickness (0.6 mm), and the largest matrix size available (512 × 512) for the EID-CT system, as well as quality iteration reconstruction level 3, were used for both scanners. The clinical EID-CT protocol consisted of a soft-tissue dedicated kernel, Br40 with iMAR, a bone kernel, Br59 with iMAR, and a slightly sharper bone kernel, Br64 (iMAR unavailable). Similar kernels were chosen for the PCD-CT system: a soft-tissue kernel, Br44, with iMAR at 70 keV and without iMAR at 110 keV, the sharpest bone kernel available with iMAR, Br56, at 70 keV, as well as the sharpest kernel available as VMI, Br76, at 70, 110, and 150 keV. 70 keV was chosen as the standard level as it corresponded to the mean energy of the 140 kV scan. An even sharper kernel, Br80, with 0.2 mm slice thickness and a 1,024 × 1,024 matrix, available only as polyenergetic “T3D”, was added to further demonstrate the PCD-CT capabilities. The sharper EID-CT Br69 kernel was considered but not used, because image quality appeared low due to excessive noise. The same field of view (FOV) was used for both scanners, ranging from 168 to 209 mm. For one patient, the FOV was accidentally set at 190 instead of 209 mm for the EID-CT Br44 iMAR and Br64 reconstructions, which was considered a minor deviation from protocol unlikely to affect the results.

#### Image evaluation

Image evaluation was conducted in two parts. First, five radiologists (M.L., 14 years subspecialized in MSK, L.T., 10 years MSK, M.C., 8 years MSK, I.K., 4 years into radiology residency, J.B., 1.5 years into radiology and 3 years orthopedic residency) individually rated 120 image stacks (12 patients × 10 reconstructions) in randomized order using the institutional research PACS (Sectra Workstation IDS7 version 19.1, Sectra AB). Axial images were shown on the left screen and multi-planar reconstructions (MPR) on the right. Preset and custom window settings were available. Image planes could be freely adjusted. Images were anonymized, and image information was blinded. A coaching session with other patients’ images preceded the evaluation. A form assessed metal artifacts, overcorrections/new artifacts, bone and soft-tissue visibility, and overall quality (Table 1). A 7-point Likert scale was used except for “Overcorrections or new artifacts” which was rated on a 5-point scale. Bone and soft tissue were rated in artifact-free areas; soft tissue was assessed at the anterior cruciate ligament (ACL) and sartorius muscle. A second reading in a different random order occurred more than five weeks later by two radiologists (J.B. and M.C.).

**Table 1:** Image evaluation criteria and interpretation of Likert scores in the first part of the evaluation

Image quality criteria			Likert scale
Metal artifacts	M1	Severity of hypodense or hyperdense streaks at the level with several screws (broad part of the plate).	1 = very severe 2 = severe 3 = marked 4 = moderate 5 = mild 6 = negligible 7 = absent 99 = unevaluable
	M2	Severity of hypodense or hyperdense streaks at the level of the distal screw.	
	M3	Severity of metal artifacts at the metal-bone interface of the distal screw	
Overcorrections of artifacts or new artifacts	OC	Overcorrections of artifacts or new artifacts: Presence and significance	1 = very significant, severely affecting diagnostic confidence 2 = significant, affecting diagnostic confidence 3 = moderate, minor effect on diagnostic confidence 4 = negligible, not affecting diagnostic confidence 5 = none 99 = unevaluable
Bone	B1	In areas not affected by metal artifacts: How is the visualization of the cortical bone?	1 = bad 2 = very poor (unacceptable) 3 = poor 4 = fair (acceptable) 5 = good 6 = very good 7 = excellent 99 = unevaluable
	B2	In areas not affected by metal artifacts: How is the visualization of the trabecular architecture?	
	B3	In areas not affected by metal artifacts: How is the visualization of the fracture and fracture fragments?	
Soft tissue	S1	In areas not affected by metal artifacts: How is the visualization of the borders of the ACL?	
	S2	In areas not affected by metal artifacts: How is the visualization of the borders of the sartorius muscle?	
Overall	O1	How is the overall image quality regarding bone?	
	O2	How is the overall image quality regarding soft tissue?	

*ACL* Anterior cruciate ligament

Second, three radiologists (M.C., L.T., and I.K.) compared images side-by-side: “new” (iMAR, VMI 110 or 150 keV) on the left *versus* “original” (without iMAR, VMI 70 keV) on the right, with other settings unchanged. Images were anonymized and blinded. MAR, overcorrections/new artifacts, and diagnostic value of “new” *versus* “original” were rated on a 5-point scale (Table S1).

#### Quantitative measurements

Noise was quantified as the standard deviation of Hounsfield Unit (HU) values within volumes of interest (VOIs). These measurements were performed across all images, as well as an additional sharper EID-CT reconstruction kernel (Br69). Three VOIs per patient were selected on the PCD CT Br44 70 keV images without iMAR by a radiologist (AB), placed in homogeneous fat while avoiding metal-related artifacts. The VOI diameter was 10 mm, reduced only when necessary to maintain tissue homogeneity (Fig. S1).

VOIs were placed in 3D Slicer [31] and transferred to all other reconstructions using rigid transformations derived from PCD-CT to EID-CT registration with the General Registration (BRAINS) module. The femur and tibia were registered separately to minimize misalignment from knee flexion. Because soft tissue compression varied between scans, all transformations were manually checked and adjusted to ensure accurate VOI alignment.

For each VOI, the mean value and standard deviation (${\sigma }_{i}$) were extracted from the software. To get a proxy for the noise level of each image reconstruction, the sum of the variances, ${\sigma }^{2}$ was calculated using the following formula:

$${\sigma }^{2}={\sum }_{i=1}^{M}\frac{{n}_{i}}{N}{\sigma }_{i}^{2}.$$

where ($\sigma$) is the sample standard deviation of the HU values, ${n}_{i}$ is the number of voxels for the VOI, and $M$ is the total number of VOIs $N={\sum }_{i=1}^{M}{n}_{i}$.

#### Statistical analysis

SPSS Statistics (version 29.0.2.0 (20) IBM SPSS Statistics) was used for reliability assessment and statistical analysis of noise measurements. Inter-reader reliability was assessed for all five readers using intra-class correlation (ICC), two-way mixed, consistency, and average measures. Intrareader reliability assessment used the ICC two-way mixed effects, absolute agreement, single measures. ICC values of < 0.40 were interpreted as poor agreement, 0.40–0.59 as fair agreement, 0.60–0.74 as good agreement, and 0.75–1.0 as excellent agreement [32]. A test of normality was performed for continuous variables (noise measurements) using the Shapiro-Wilk test, and as normality could not be assumed, comparisons of noise levels were done using Wilcoxon’s signed rank test.

The VGC Analyzer software [33] was used for visual grading characteristics (VGC) analysis of qualitative data. VGC is developed for non-parametric analysis of visual grading data and compares the image quality ratings of two conditions. The statistical basis of VGC analysis is similar to the receiver operating characteristic (ROC) analysis, with the difference that independence of cases is not assumed. The input values are the raw scores given by readers. The settings “paired data” and “random readers” were used. The area under the curve (AUC) is reported along with the 95% confidence interval and *p*-value. AUC values > 0.5 indicate superior image quality for the test image, whereas values < 0.5 indicate superior quality for the reference image. *p*-values ≤ 0.05 were considered statistically significant. As the VGC analyzer demands fully crossed data, one missing value was imputed using the mean of the other readers’ scores for the same patient and image reconstruction.

## Results

### Qualitative evaluation

The mean scores from the visual grading assessments are summarized in Table 2 and the frequencies of scores in Tables S2 and 3. Two readers misunderstood the questions regarding overall image quality, thinking it referred only to areas without metal artifacts, which was not the intention, and their results on these questions were omitted from the analysis.

**Table 2:** Mean visual grading scores across patients and readers for each reconstruction setting and quality criterion

The heatmap colors represent these scores, with red and green corresponding to scores of 2 and 6, respectively. Scores below or above these thresholds are displayed using the same colors as the threshold values

*PCD-CT* Photon-counting CT, *EID-CT* Energy integrating CT, i*MAR* Iterative metal artifact reduction, *ACL* Anterior cruciate ligament

#### Metal

**Fig. 1:**
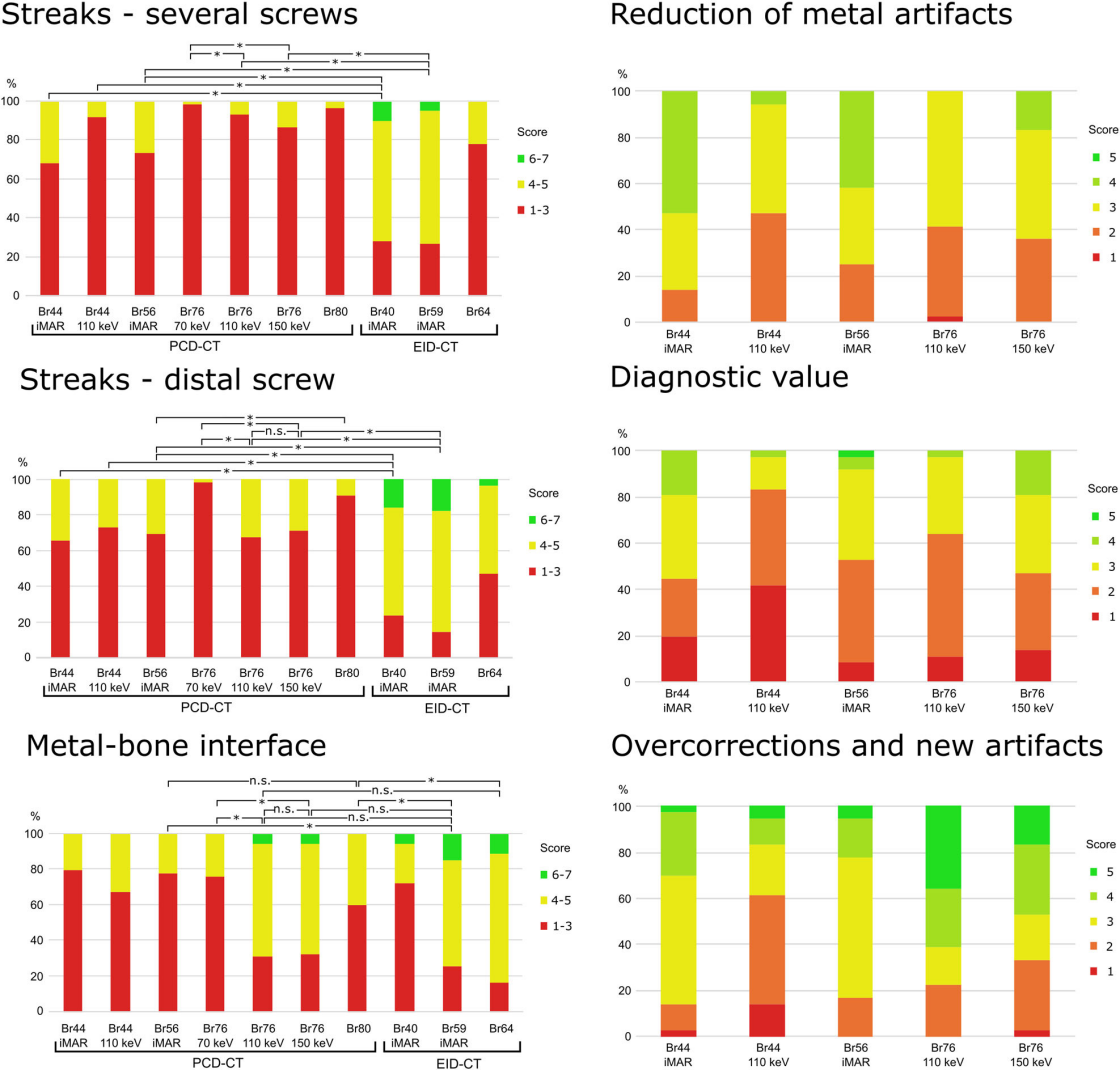
Percentage distribution of observer scores for metal artifact image quality criteria. Stacked bar charts illustrate the distribution of observer scores for image quality criteria related to metal artifacts, with frequencies expressed as percentages of the total number of ratings. Comparisons of scores performed using VGC analysis are indicated by brackets, where * denotes a statistically significant difference and n.s. a non-significant difference. The left pane shows assessments of streaks at the level of several screws, streaks at the level of the distal screw, and the metal–bone interface. As one patient lacked a distal screw, images from 11 rather than 12 patients were included for the latter two criteria. The right pane presents results from the side-by-side evaluation, in which each image type was compared with a reference image without iMAR and with VMI 70 keV, evaluating the reduction of metal artifacts, the added diagnostic value, and the significance of overcorrections or newly introduced artifacts

**Fig. 2:**
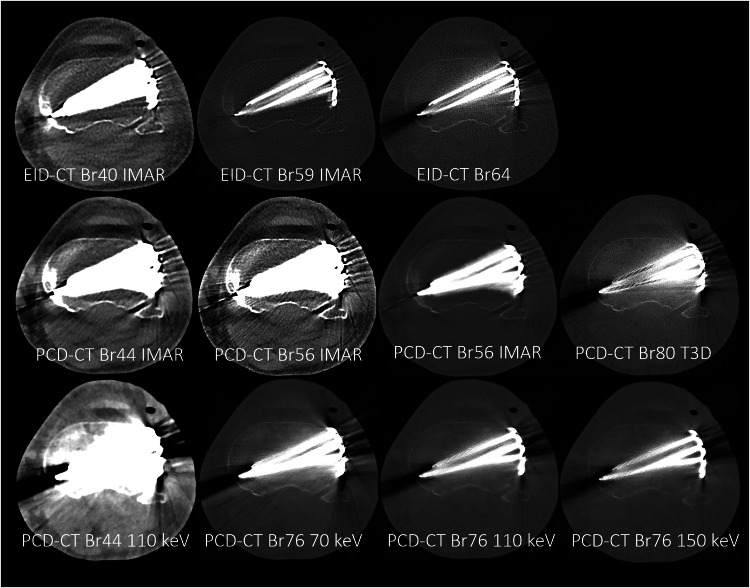
Representative axial CT images of a fixation plate and screws at the proximal tibia. iMAR reconstruction markedly reduces streak artifacts, though small hyperdense areas remain adjacent to screws. The top row displays EID-CT images, while the middle and bottom rows show PCD-CT images. Window settings are optimized for the intended use, with soft-tissue evaluation shown on the left and bone evaluation on the right

**Fig. 3:**
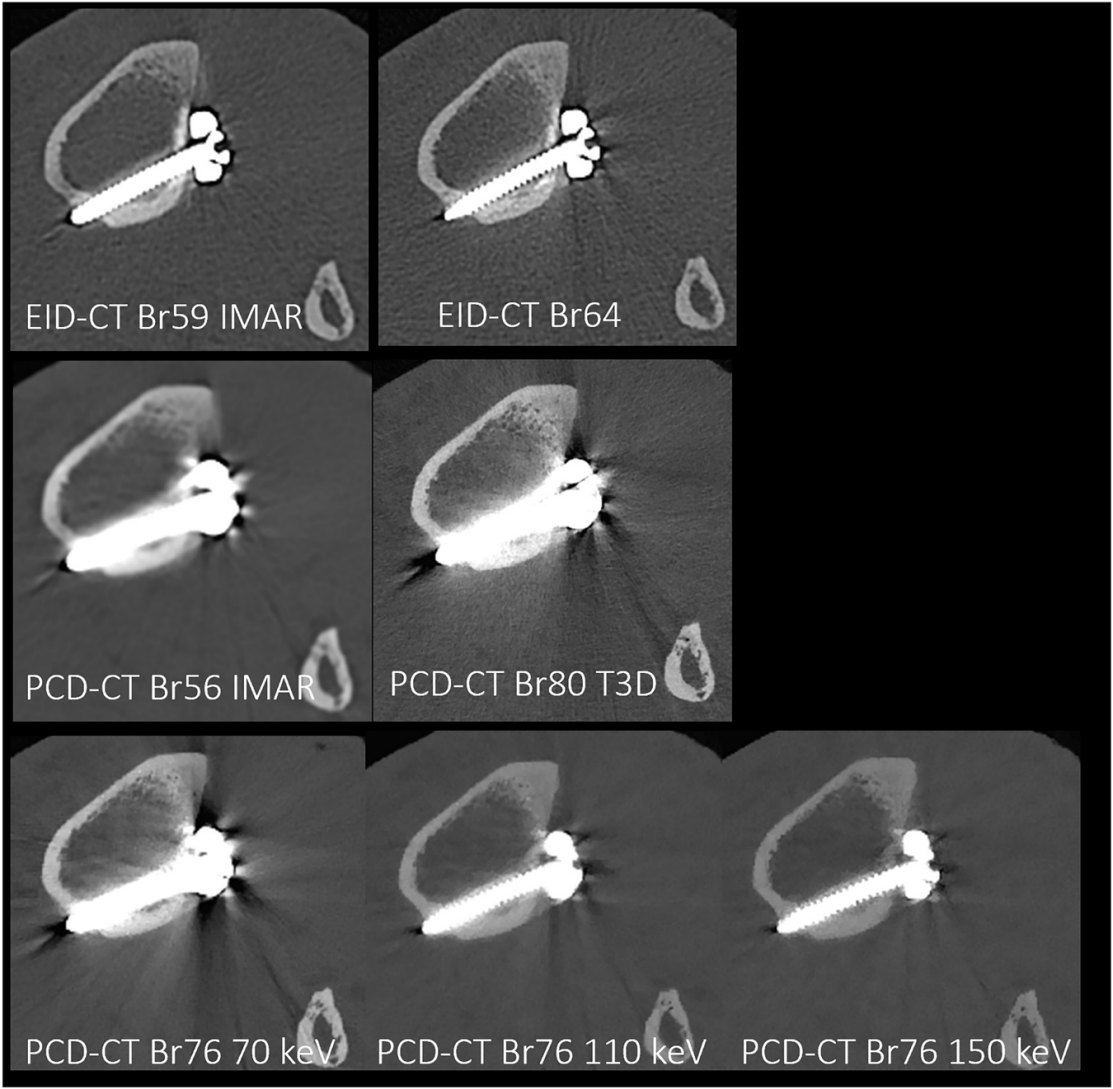
Representative axial CT images showing the metal-bone interface for a screw in the tibia. The top row displays EID-CT images, while the middle and bottom rows show PCD-CT images

In one patient, only screws were placed at the level of the tibial plateau, without an accompanying plate. Consequently, image quality criteria M2–M3 related to the distal screw could not be assessed. The percentages of scores for each criterion are shown in Fig. 1. EID-CT Br59 iMAR images were rated higher than PCD-CT Br56 iMAR, Br76 110 keV, and 150 keV images for both the level of several screws (Fig. 2) and the level of the distal screw (AUC 0.13–0.16). For the metal-bone interface, no difference was found between EID-CT Br59 iMAR images and PCD-CT Br76 110 keV and 150 keV images, respectively (AUC 0.42–0.49) (Fig. 3). EID-CT Br40 iMAR images were rated higher, compared to both PCD-CT Br44 iMAR and Br44 110 keV images (AUC 0.10–0.21). Table S4 summarizes the results from the VGC analysis.

PCD-CT images were also compared side-by-side (Fig. 1). For sharp kernels, iMAR was considered to provide high to very high reduction of metal artifacts in 42% of ratings, whereas VMI 110 keV and 150 keV did so in 0% and 17% of ratings, respectively. The difference between 110 and 150 keV was not statistically significant (AUC 0.58, *p* = 0.247). The diagnostic value of iMAR, VMI 110 keV and 150 keV in bone kernels was rated as high or very high in 8, 3, and 19% of ratings, respectively. There was no statistically significant difference between these image types. The smooth kernel Br44 with iMAR was rated superior to VMI 110 keV with 53% of iMAR ratings indicating substantial to almost complete reduction compared to 6% for VMI 110 keV (AUC 0.21, *p* = 0.006). The diagnostic value of these reconstructions was considered higher in iMAR images compared to VMI 110 keV, with the rating of high to very high in 19% of cases compared to 3% (AUC 0.23, *p* = 0.005).

**Fig. 4:**
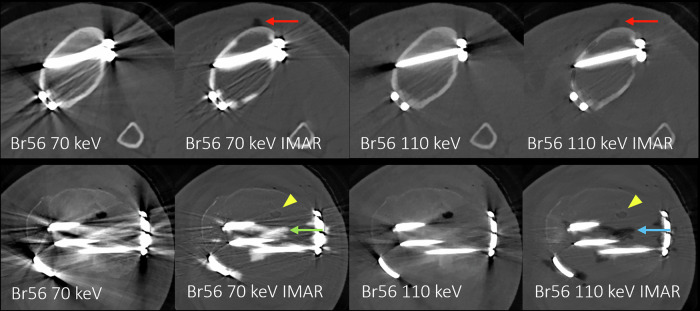
Axial CT images showing artifacts and loss of anatomical information in iMAR and high-VMI images. Images were reconstructed using a Br56 kernel with 0.4 mm slice thickness, with and without iMAR at 70 and 110 keV. A low-attenuating artifact adjacent to the tibia appears (red arrow), and a gas bubble within the tibia is almost eliminated (yellow arrowhead) in iMAR images. Hyperattenuating artifacts are enhanced at 70 keV with iMAR (green arrow) and are converted into low-attenuating artifacts at 110 keV with iMAR (blue arrow)

The presence of overcorrections or new artifacts, exemplified in Fig. 4, was observed in more than 90% of ratings for Br44 iMAR, Br44 110 keV and Br56 iMAR. For Br76 110 keV and 150 keV this was noted in 44% and 83% of ratings, respectively. In two instances, readers denied the presence of overcorrections or new artifacts but nonetheless rated their significance as 4 instead of 5; these inconsistencies were corrected in Table S3 and the subsequent analyses. Overcorrections or new artifacts were considered significant or very significant in 61% of ratings for Br44 110 keV and in 34% for Br76 150 keV, whereas lower proportions were observed for Br76 110 keV (22%), Br56 iMAR (17%), and Br44 iMAR (14%), in descending order.

#### Bone

Bone visualization was rated similarly between the iMAR images from the EID-CT and the PCD-CT, with mean scores for the three bone visualization criteria ranging from 4.6 to 5.3 for EID-CT and from 4.8 to 5.4 for PCD-CT (AUC 0.53, 0.59, and 0.53, respectively). Mean scores of the sharper EID-CT Br64 and PCD-CT Br76 70 keV reconstruction were also rated similarly, and VGC analysis was not performed. To demonstrate the additional capabilities of the PCD-CT, a sharper kernel, Br80, was reconstructed with a thinner 0.2 mm slice thickness and a larger 1024 × 1024 matrix. Readers rated this reconstruction significantly higher than the EID-CT Br64 reconstruction in all three bone criteria (AUC 0.80, *p* = 0.004, AUC 0.91, *p* = 0.000, and AUC 0.79, *p* = 0.004, respectively). The criterion for overall bone visualization, which includes areas with and without metal artifacts, also showed Br80 to be superior to EID-CT Br56 iMAR and Br64 (AUC 0.72 and 0.76, respectively).

**Fig. 5:**
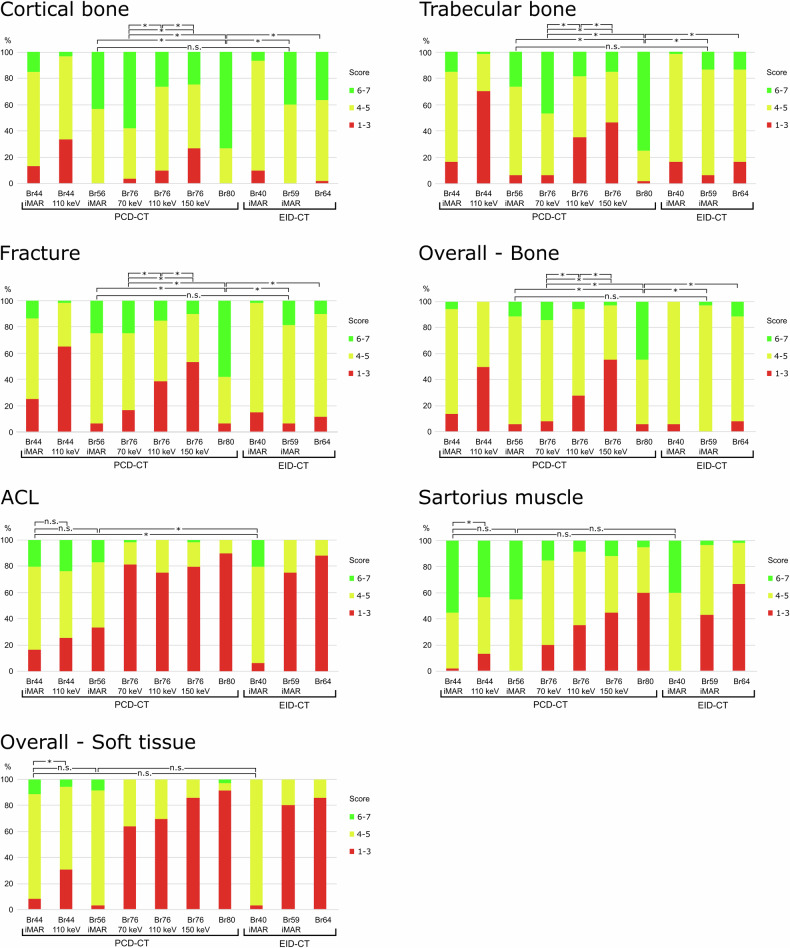
Percentage distribution of observer scores for bone and soft-tissue image quality criteria. Stacked bar charts illustrate the distribution of observer scores for image quality criteria related to bone and soft tissue. Frequencies are expressed as percentages of the total number of ratings. Comparisons of scores performed using VGC analysis are indicated by brackets, where * denotes a statistically significant difference and n.s. a non-significant difference

**Fig. 6:**
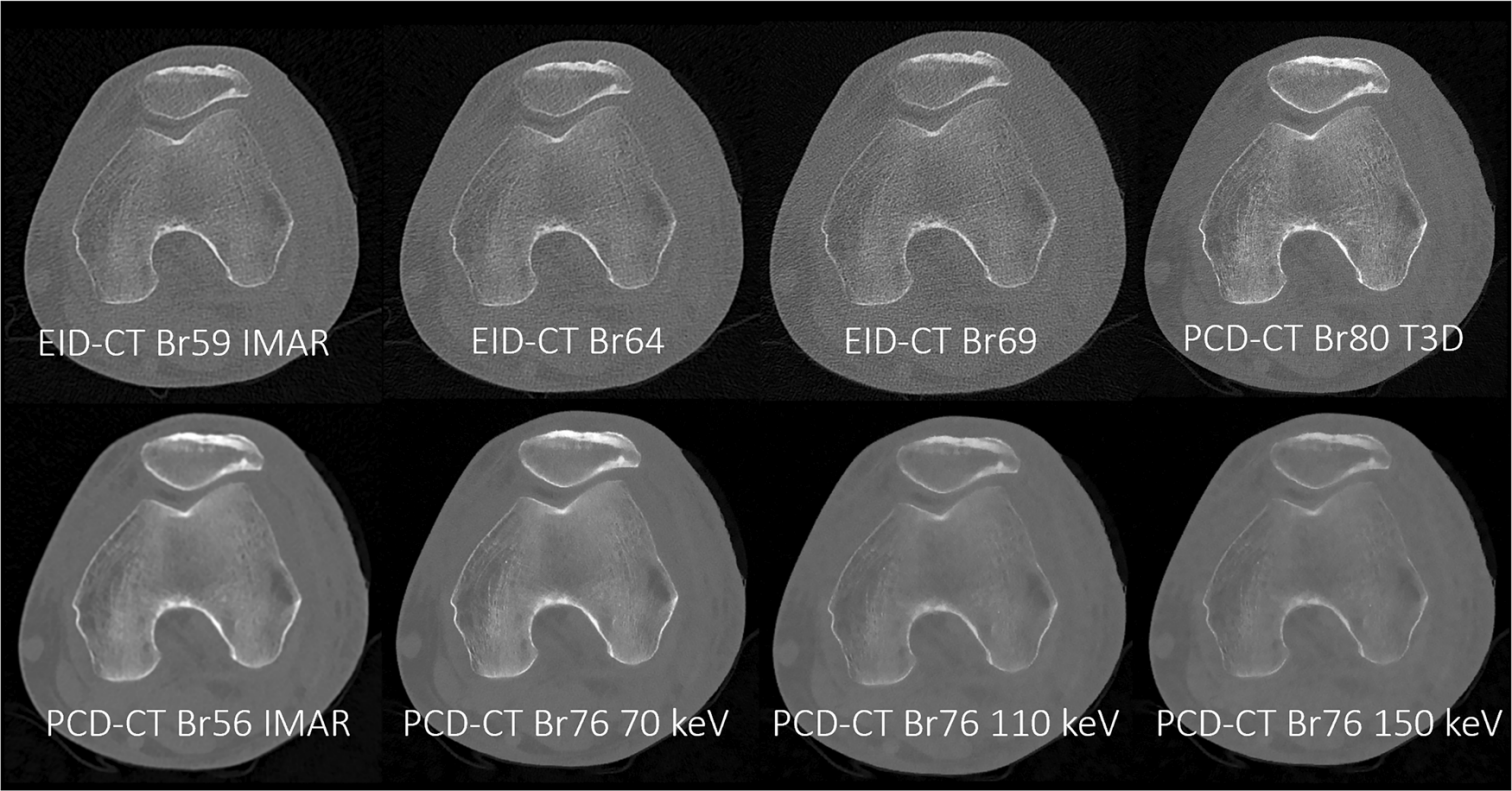
Representative axial CT images of sharp kernels centered over the distal femur

Radiologists attributed higher scores for bone visualization to the standard PCD-CT Br76 70 keV, with mean scores ranging from 4.7–5.6 compared to 4.1–4.2 and 3.7–4.5 for 110 keV and 150 keV, respectively. The differences were statistically significant (AUC values ranging from 0.13 to 0.32 and *p*-values ≤ 0.009). Figure 5 demonstrates the frequencies of scores for all bone quality criteria, and Table S5 presents the results from the VGC analysis. Representative images from each of the sharp kernel reconstructions are shown in Fig. 6.

#### Soft tissue

The extraarticular sartorius muscle and the overall scores for soft tissues were rated similarly for both scanners, whereas for the intraarticular ACL, there was a slight preference for the EID-CT Br40 images compared to both the PCD-CT Br44 and the semi-sharp Br56 images (AUC 0.38, *p* = 0.049 and AUC 0.29, *p* = 0.006, respectively) (Fig. 5 and Table S6). Figure S2 demonstrates representative images of the soft-tissue kernels compared. Intercomparison of PCD-CT images yielded a small preference for lower keV for visualization of the sartorius muscle and overall soft tissue (AUC 0.37, *p* = 0.038 and AUC 0.31, *p* = 0.024, respectively), whereas no clear preference was found for visualization of the ACL.

##### Inter- and intrareader reliability

The mean ICC of the inter-reader analysis was 0.83 (range 0.75–0.89), indicating excellent agreement. Intrareader reliability average was 0.48 (range 0.41–0.54) for the less experienced reader and 0.66 (range 0.46–0.77) for the more experienced one, indicating fair to good agreement (Table 3).

**Table 3:** Results from the inter- and intra-reader reliability analysis of reader scores across image quality criteria

Image criteria	Interreader		Intrareader: Reader 1		Intrareader: Reader 2	
	ICC	95% CI	ICC	95% CI	ICC	95% CI
M1	0.84	0.80–0.88	0.54	0.41–0.66	0.63	0.35–0.78
M2	0.83	0.77–0.87	0.49	0.34–0.62	0.63	0.49–0.74
M3	0.75	0.67–0.82	0.51	0.36–0.64	0.46	0.29–0.60
B1	0.76	0.72–0.84	0.42	0.23–0.58	0.70	0.60–0.78
B2	0.85	0.81–0.89	0.51	0.20–0.69	0.69	0.58–0.77
B3	0.80	0.74–0.85	0.41	0.23–0.55	0.70	0.59–0.78
S1	0.89	0.85–0.92	0.47	0.32–0.60	0.77	0.66–0.84
S2	0.86	0.82–0.90	0.45	0.27–0.60	0.63	0.41–0.76
O1	0.79	0.73–0.85	0.46	0.29–0.60	0.72	0.62–0.80
O2	0.89	0.85–0.92	0.49	0.33–0.62	0.69	0.58–0.77
Mean	0.83		0.48		0.66	

ICC with corresponding 95% confidence interval (CI)

*M1* Metal artifact streaks at the level of several screws, *M2* Metal artifact streaks at the level of the distal screw, *M3* Metal-bone interface, *B1* Cortical bone, *B2* Trabecular architecture, *B3* Fracture, *S1* Anterior cruciate ligament, *S2* Sartorius muscle, *O1* Overall bone, *O2* Overall soft tissue

### Quantitative measurements

**Fig. 7:**
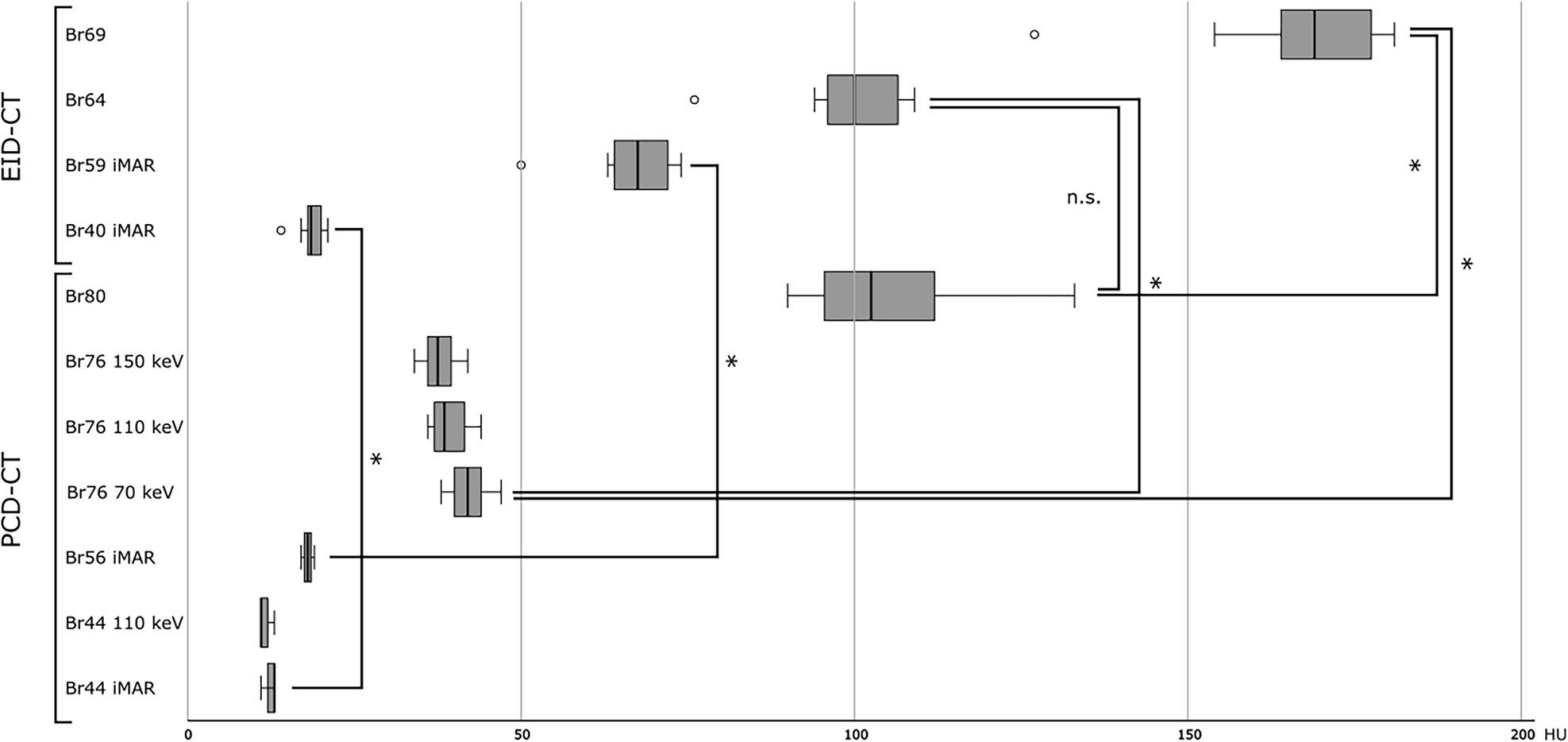
Box plots of noise levels. Results for all images included in the qualitative evaluation, as well as for the sharper EID-CT Br69 kernel, are shown. Noise levels were compared using Wilcoxon’s signed-rank test, with * indicating a statistically significant difference, and n.s. a non-significant difference. The median is shown as a horizontal line. The box represents the interquartile range (IQR), spanning the first to third quartiles, and the whiskers extend to 1.5 times the IQR. Outliers are marked with °

Noise levels increased with the sharpness of the kernel (Fig. 7). The lowest noise was observed for the PCD-CT Br44 (12 HU) and the EID-CT Br40 (18 HU). Noise levels were generally higher for EID-CT images than for the corresponding PCD-CT images. Especially high noise levels were noted in Br69 (167 HU), which was not included in the qualitative image evaluation. The noise level of the PCD-CT Br80 (104 HU), although reconstructed in a thinner slice thickness (0.2 mm), was similar to that of the EID-CT Br64 (100 HU).

## Discussion

This study compares the severity of metal artifacts and the visualization of bone and soft tissue in postoperative knee images acquired on PCD-CT and EID-CT. Metal artifact streaks were less severe in EID-CT iMAR images compared to PCD-CT iMAR and 110/150 keV images, whereas the metal-bone interface was rated similarly for EID-CT iMAR and PCD-CT 110/150 keV images. The side-by-side comparison of PCD-CT images demonstrated the high efficacy of iMAR, whereas VMI 110/150 keV showed only moderate artifact reduction. Bone visualization was superior in the sharpest PCD-CT kernel (Br80). ACL visualization was slightly better for EID-CT, whereas scores were almost equal for the sartorius muscle. Overall image quality of the PCD-CT for both bone and soft tissues was higher for low VMI compared to high VMI. Noise was generally lower in PCD-CT images compared to EID-CT images with similar kernels, especially for sharper kernels.

Advantages of PCD-CT in MSK imaging have mainly been linked to improved spatial resolution and reduced metal artifacts [34], but evidence for the latter is limited, as most studies compare PCD-CT settings internally rather than to EID-CT. One study compared PCD-CT polyenergetic images, VMI 130 keV and EID-CT images for patients with wrist prostheses. Streak artifacts improved with PCD-CT 130 keV *versus* polyenergetic images, but not compared to EID-CT. The metal-bone interface was best visualized with PCD-CT polyenergetic images and EID-CT images [35], contrasting with our findings where high VMI scored higher—likely due to implant material differences, as titanium is less problematic than stainless steel, making resolution benefits outweigh theoretical artifact reduction. The results from the present study confirm previous findings for hip prostheses [20] and dental implants [10, 19, 36, 37] in which the use of high VMI alone was much less effective than iMAR. This limitation is likely attributable to photon starvation caused by stainless steel and other high-atomic-number metals [38, 39]. In contrast, titanium, which has a lower atomic number, primarily induces artifacts related to beam hardening rather than photon starvation. In such cases, VMI might be sufficient, as demonstrated in studies of spinal and ankle fixation showing effective artifact reduction using VMI at 110–120 keV [11, 16]. The MAR algorithm currently available for the PCD-CT was less efficient than for the EID-CT. Future algorithmic developments leveraging the spectral information inherently provided by PCD-CT systems [38] may further improve MAR.

The combination of iMAR and high VMI has been reported to be advantageous for MAR [7, 20, 36] but has also been associated with the emergence of new artifacts [20, 40]. During the preparatory phase of the present study, such artifacts were observed (Fig. 4), which led us to exclude these reconstructions from the image quality evaluation.

Various methods for artifact quantification are commonly employed; however, their correlation with subjective image scores is low, and reproducibility is limited [2]. Both segmentation [7, 41] and Fourier analysis [35, 42] were considered in the present study but deemed unsuitable: segmentation proved infeasible due to difficulties in defining an appropriate threshold, and Fourier analysis is poorly suited for irregular structures. Since image quality is ultimately determined by the interpreting radiologist, qualitative ratings are regarded as the gold standard [2].

Overcorrections and new artifacts in iMAR and VMI have been reported previously [17, 40, 43], which is concordant with the high frequency of such findings in the side-by-side comparison of PCD-CT images. However, in the first part of the image evaluation, such findings were also indicated in uncorrected images, likely reflecting difficulty distinguishing such phenomena from true artifacts. This was confirmed by one reader after becoming familiar with the phenomenon during side-by-side review. This justified excluding such data emanating from the first part of the image evaluation from further analysis. The finding also underscores the value of a reference image when rating such criteria.

Sharper kernels inherently amplify noise, and in bone imaging, there is always a trade-off between choosing a sharp kernel and accepting more noise. For the PCD-CT, however, sharper kernels are often well tolerated and even preferred [35, 44, 45]. To demonstrate the additional capabilities of the PCD-CT, we added a very sharp kernel (Br80) with a higher matrix and thinner slices. Noise was indeed high, but it was still rated the highest for visualization of bone structures, both in regions unaffected by metal artifacts and for the overall bone criterion that included artifact‑affected areas, which is in line with previous studies [44]. This suggests that although metal artifacts were more pronounced in the PCD‑CT images, the superior spatial resolution of the Br80 reconstruction may still make it preferable to less artifact‑affected images with lower bone detail.

Concerning soft tissues, the results showed a preference for EID‑CT over PCD‑CT in the visualization of the ACL, for which we found no clear explanation. The ACL is not located close enough to the hardware for streak artifacts to be a likely cause, and the other soft‑tissue criteria were rated similarly between scanners, suggesting that this finding may reflect chance variation. Another observation is the comparable soft‑tissue scores for Br44 iMAR and Br56 iMAR, likely related to the lower noise levels in PCD‑CT images. This may support the use of slightly sharper kernels, such as Br56, for soft‑tissue assessment on PCD‑CT.

To our knowledge, this is the first study reporting on the reduction of metal artifacts in patients’ knees using PCD-CT, as well as the first study comparing it to EID-CT. Comparisons of metal artifacts between PCD-CT and EID-CT are scarce, as well as using images from real clinical cases, not phantoms or cadavers. Quantifying metal artifacts has proven difficult, and there is poor correlation with visual assessment [2]. In this study, the conclusions are drawn from qualitative evaluation by five radiologists using a statistical method specifically adapted for visual grading data. A limitation of the study is the small study group, a consequence of challenges in patient recruitment due to short hospital stays and limited scanner availability. Intra-reader reliability was only fair to good, and additional prior training might have improved consistency. However, five readers participated, and inter-reader reliability was excellent, supporting the robustness of the overall assessment.

To conclude, in this first *in vivo* comparison of PCD-CT and EID-CT for postoperative knee imaging with metallic osteosynthesis material, PCD-CT iMAR and high VMI were rated inferior to EID-CT iMAR images for overall MAR, except at the metal–bone interface, where high-energy VMIs performed comparably. These findings indicate that further improvements in MAR algorithms for PCD-CT are warranted. PCD-CT demonstrated a clear advantage over EID-CT in bone visualization, particularly with the ultra-high resolution Br80 reconstruction, which was rated superior even when artifact-affected regions were included. Together, these results provide clinically relevant guidance for postoperative knee imaging and support a complementary reading strategy combining ultra-high-resolution images, high-energy VMI, and MAR-corrected images in PCD-CT.

## Supplementary information

**Additional File 1:** **Table S1**. Image evaluation criteria and interpretation of Likert scores in the second part of the evaluation. **Table S2**. Frequency table of scores from the first part of the image quality evaluation. **Table S3**. Frequencies and percentages of observer scores for the side-by-side comparison of image reconstruction types compared to the corresponding reference reconstruction (no iMAR, 70 keV). **Table S4**. Results of the VGC analysis for image criteria concerning metal artifacts. **Table S5**. Results of the VGC analysis for image criteria concerning bone. **Table S6**. Results of the VGC analysis for image criteria concerning soft tissue for each compared pair of image types. **Fig. S1**. Representative example of VOI placement. **Fig. S2**. Representative axial CT images of smooth-kernel reconstructions at the level of the distal femur.

## Data Availability

The datasets used and/or analyzed during the current study are available from the corresponding author on reasonable request. Restrictions may apply due to licensing and/or regulatory reasons.

## Abbreviations

ACL: Anterior cruciate ligament
AUC: Area under the curve
EID-CT: Energy-integrating detector
ICC: Intra-class correlation
iMAR: Iterative metal artifact reduction
MAR: Metal artifact reduction
MSK: Musculoskeletal
PCD-CT: Photon-counting detector
VGC: Visual grading characteristics
VMI: Virtual monoenergetic images
