# Supplementary material for: Photon-counting CT: image quality evaluation in patients with tibial plateau fracture treated with metallic osteosynthesis material
Source: Eur Radiol Exp. 2026 Jun 19;10:96. doi: 10.1186/s41747-026-00761-8 (PMC13282422; doi:10.1186/s41747-026-00761-8)

Photon-counting CT: Image quality evaluation in patients with tibial plateau fracture treated with metallic osteosynthesis material

ELECTRONIC SUPPLEMENTARY MATERIAL

Table S1 Image evaluation criteria and interpretation of Likert scores in the second part of the evaluation.

<table>
<tr><th colspan="2">Image quality criteria</th><th>Likert scale</th></tr>
<tr><td>Reduction of metal artifacts</td><td>The reduction of metal artifacts</td><td>1 = No apparent reduction
2 = Minimal
3 = Moderate
4 = Substantial
5 = Complete or almost complete

99 = unevaluable</td></tr>
<tr><td rowspan="2">Overcorrections of artifacts or new artifacts</td><td>Presence of overcorrections of artifacts or new artifacts</td><td>1 = Yes
2 = No

99 = unevaluable</td></tr>
<tr><td>The significance of overcorrections of artifacts or new artifacts is:</td><td>1 = very significant, severely affecting diagnostic confidence
2 = significant, affecting diagnostic confidence
3 = moderate, minor effect on diagnostic confidence
4 = negligible, not affecting diagnostic confidence
5 = none

99 = unevaluable</td></tr>
<tr><td>Diagnostic value</td><td>The diagnostic value of the added information</td><td>1 = None or no added information
2 = Low
3 = Moderate
4 = High
5 = Very high

99 = unevaluable</td></tr>
</table>

Table S2 Frequency table of scores from the first part of the image quality evaluation.

				**Metal artifacts**																				
				**Several screws (M1)**							**Single screw (M2)**							**Metal-bone interface (M3)**						
**Scanner**	**Kernel**	**keV**	**iMAR**	**1**	**2**	**3**	**4**	**5**	**6**	**7**	**1**	**2**	**3**	**4**	**5**	**6**	**7**	**1**	**2**	**3**	**4**	**5**	**6**	**7**
	**Br44**	**70**	**iMAR**	7	11	23	15	4	0	0	2	11	23	9	10	0	0	7	23	14	9	2	0	0
	**Br44**	**110**		12	25	18	5	0	0	0	8	15	17	14	1	0	0	12	14	11	15	3	0	0
	**Br56**	**70**	**iMAR**	8	14	22	14	2	0	0	7	7	24	10	7	0	0	6	13	24	11	1	0	0
**PCD-CT**	**Br76**	**70**		26	31	2	1	0	0	0	20	23	11	1	0	0	0	4	15	23	8	5	0	0
	**Br76**	**110**		7	24	25	3	1	0	0	3	14	20	13	5	0	0	0	4	13	16	19	3	0
	**Br76**	**150**		15	20	17	6	2	0	0	7	9	23	11	5	0	0	1	8	9	21	13	3	0
	**Br80**	**T3D**		13	25	20	1	1	0	0	10	16	24	4	1	0	0	3	10	20	16	6	0	0
	**Br40**		**iMAR**	1	5	11	21	16	6	0	1	2	10	12	21	9	0	1	16	23	10	2	3	0
**EID-CT**	**Br59**		**iMAR**	0	6	10	21	20	3	0	0	1	7	14	23	10	0	0	0	14	24	9	8	0
	**Br64**			1	19	27	9	4	0	0	0	10	16	21	6	2	0	0	1	8	19	21	6	0

				**Bone**																				
				**Cortical bone (B1)**							**Trabecular bone (B2)**							**Fracture (B3)**						
**Scanner**	**Kernel**	**keV**	**iMAR**	**1**	**2**	**3**	**4**	**5**	**6**	**7**	**1**	**2**	**3**	**4**	**5**	**6**	**7**	**1**	**2**	**3**	**4**	**5**	**6**	**7**
**PCD-CT**	**Br44**	**70**	**iMAR**	0	1	7	18	25	7	2	0	1	9	33	8	8	1	0	1	14	29	8	7	1
	**Br44**	**110**		0	5	15	29	9	2	0	1	15	26	14	3	0	1	3	13	23	13	7	0	1

	**Br56**	**70**	**iMAR**	0	0	0	5	29	24	2	0	0	4	20	20	12	4	0	0	4	14	27	12	3
	**Br76**	**70**		0	0	2	8	15	21	14	0	0	4	10	18	17	11	0	1	9	15	20	10	5
	**Br76**	**110**		0	0	6	17	21	10	6	1	3	17	17	11	6	5	0	3	20	17	11	6	3
	**Br76**	**150**		0	2	14	15	14	12	3	0	16	12	17	6	5	4	0	7	25	14	8	5	1
	**Br80**	**T3D**		0	0	0	4	12	18	26	0	1	0	4	10	23	22	0	1	3	9	12	21	14
	**Br40**		**iMAR**	0	0	6	26	24	2	2	1	1	8	38	11	1	0	0	0	9	26	24	1	0
**EID-CT**	**Br59**		**iMAR**	0	0	0	10	26	21	3	0	0	4	26	22	8	0	0	0	4	19	26	8	3
	**Br64**			0	0	1	6	31	19	3	0	0	10	19	23	6	2	0	0	7	19	28	6	0

				**Soft tissue**													
				**Anterior cruciate ligament (S1)**							**Sartorius muscle (S2)**						
**Scanner**	**Kernel**	**keV**	**iMAR**	**1**	**2**	**3**	**4**	**5**	**6**	**7**	**1**	**2**	**3**	**4**	**5**	**6**	**7**
	**Br44**	**70**	**iMAR**	0	4	6	26	12	11	1	0	0	1	7	19	18	15
	**Br44**	**110**		2	5	8	16	14	10	4	0	3	5	16	10	11	15
	**Br56**	**70**	**iMAR**	0	5	15	20	10	10	0	0	0	0	8	25	13	14
**PCD-CT**	**Br76**	**70**		5	18	25	6	4	1	0	0	2	10	24	15	9	0
	**Br76**	**110**		3	15	27	14	1	0	0	1	6	14	22	12	5	0
	**Br76**	**150**		6	21	21	9	2	1	0	3	8	16	16	10	5	2
	**Br80**	**T3D**		20	22	12	4	2	0	0	2	15	19	15	6	3	0
**EID-CT**	**Br40**		**iMAR**	0	2	2	22	22	7	5	0	0	0	14	22	11	13

	**Br59**		**iMAR**	7	18	20	13	2	0	0	1	6	19	24	8	2	0
	**Br64**			19	23	11	5	2	0	0	5	10	25	14	5	1	0
				**Overall**													
				**Bone (O1)**							**Soft tissue (O2)**						
**Scanner**	**Kernel**	**keV**	**iMAR**	**1**	**2**	**3**	**4**	**5**	**6**	**7**	**1**	**2**	**3**	**4**	**5**	**6**	**7**
	**Br44**	**70**	**iMAR**	0	0	5	17	12	2	0	0	0	3	15	14	4	0
	**Br44**	**110**		0	5	13	16	2	0	0	0	4	7	15	8	2	0
	**Br56**	**70**	**iMAR**	0	0	2	9	21	4	0	0	0	1	22	10	3	0
**PCD-CT**	**Br76**	**70**		0	0	3	10	18	5	0	0	8	15	10	3	0	0
	**Br76**	**110**		0	1	9	19	5	2	0	0	8	17	11	0	0	0
	**Br76**	**150**		0	2	18	12	3	1	0	2	15	14	2	3	0	0
	**Br80**	**T3D**		0	0	2	6	12	14	2	5	19	9	2	0	1	0
	**Br40**		**iMAR**	0	0	2	17	17	0	0	0	0	1	17	18	0	0
**EID-CT**	**Br59**		**iMAR**	0	0	0	14	21	1	0	0	12	17	7	0	0	0
	**Br64**			0	0	3	16	13	4	0	2	22	7	4	1	0	0

Table S3 Frequencies and percentages of observer scores for the side-by-side comparison of image reconstruction types compared to the corresponding reference reconstruction (no iMAR, 70 keV).

					**Reduction of metal artifacts**				
**Kernel**	**VMI (keV)**	**iMAR**			**1**	**2**	**3**	**4**	**5**
Br44	70	iMAR			0 (0%)	5 (14%)	12 (33%)	19 (53%)	0 (0%)
Br44	110	no			0 (0%)	17 (47%)	17 (47%)	2 (6%)	0 (0%)
Br56	70	iMAR			0 (0%)	9 (25%)	12 (33%)	15 (42%)	0 (0%)
Br76	110	no			1 (3%)	14 (39%)	21 (58%)	0 (0%)	0 (0%)
Br76	150	no			0 (0%)	13 (36%)	17 (47%)	6 (17%)	0 (0%)
					**Diagnostic value**				
**Kernel**	**VMI (keV)**	**iMAR**			**1**	**2**	**3**	**4**	**5**
Br44	70	iMAR			7 (19%)	9 (25%)	13 (36%)	7 (19%)	0 (0%)
Br44	110	no			15 (42%)	15 (42%)	5 (14%)	1 (3%)	0 (0%)
Br56	70	iMAR			3 (8%)	16 (44%)	14 (39%)	2 (6%)	1 (3%)
Br76	110	no			4 (11%)	19 (53%)	12 (33%)	1 (3%)	0 (0%)
Br76	150	no			5 (14%)	12 (33%)	12 (33%)	7 (19%)	0 (0%)
			**Presence and significance of overcorrections and new artifacts**						
**Kernel**	**VMI (keV)**	**iMAR**	**Yes**	**No**	**1**	**2**	**3**	**4**	**5**
Br44	70	iMAR	35 (97%)	1 (3%)	1 (3%)	4 (11%)	20 (56%)	10 (28%)	1 (3%)
Br44	110	no	34 (94%)	2 (6%)	5 (14%)	17 (47%)	8 (22%)	4 (11%)	2 (6%)

Br56	70	iMAR	34 (94%)	2 (6%)	0 (0%)	6 (17%)	22 (61%)	6 (17%)	2 (6%)
Br76	110	no	23 (64%)	13 (36%)	0 (0%)	8 (22%)	6 (17%)	9 (25%)	13 (36%)
Br76	150	no	30 (83%)	6 (17%)	1 (3%)	11 (31%)	7 (19%)	11 (31%)	6 (17%)

Scores and percentages of scores for each image quality criteria for the side-by-side comparison. Each image was compared to the corresponding reference image without iMAR at 70 keV.
VMI Virtual Monoenergetic Images, *iMAR* iterative Metal Artefact Reduction

Table S4 Results of the VGC analysis for image criteria concerning metal artifacts.

Image criteria	Intended use	Reference image	Test image	Number of cases	AUC	95% CI	p-value	Sig.
M1	Soft tissue	**EID-CT Br40 iMAR**	PCD-CT Br44 iMAR	12	0.21	0.09 – 0.35	0.004	*
M1		**EID-CT Br40 iMAR**	PCD-CT Br44 110 keV	12	0.10	0.03 – 0.19	0.001	*
M1		**EID-CT Br40 iMAR**	PCD-CT Br56 iMAR	12	0.17	0.07 – 0.29	0.001	*
M2		**EID-CT Br40 iMAR**	PCD-CT Br44 iMAR	11	0.21	0.09 – 0.33	0.006	*
M2		**EID-CT Br40 iMAR**	PCD-CT Br44 110 keV	11	0.14	0.03 – 0.29	0.004	*
M2		**EID-CT Br40 iMAR**	PCD-CT Br56 iMAR	11	0.17	0.06 – 0.30	0.003	*
M1	Bone	**EID-CT Br59 iMAR**	PCD-CT Br56 iMAR	12	0.16	0.07 – 0.28	0.003	*
M1		**EID-CT Br59 iMAR**	PCD-CT Br76 110 keV	12	0.13	0.02 – 0.29	0.001	*
M1		**EID-CT Br59 iMAR**	PCD-CT Br76 150 keV	12	0.13	0.01 – 0.33	0.002	*
M1		**PCD-CT Br56 iMAR**	PCD-CT Br80	12	0.30	0.11 – 0.50	0.014	*
M1		PCD-CT Br76 70 keV	**PCD-CT Br76 110 keV**	12	0.82	0.70 – 0.92	0.000	*
M1		PCD-CT Br76 70 keV	**PCD-CT Br76 150 keV**	12	0.72	0.59 – 0.86	0.008	*
M2		**EID-CT Br59 iMAR**	PCD-CT Br56 iMAR	11	0.10	0.02 – 0.22	0.001	*
M2		**EID-CT Br59 iMAR**	PCD-CT Br76 110 keV	11	0.15	0.02 – 0.35	0.002	*
M2		**EID-CT Br59 iMAR**	PCD-CT Br76 150 keV	11	0.15	0.02 – 0.37	0.003	*
M2		**PCD-CT Br56 iMAR**	PCD-CT Br80	11	0.32	0.12 – 0.55	0.030	*
M2		PCD-CT Br76 70 keV	**PCD-CT Br76 110 keV**	11	0.88	0.80 – 0.95	0.001	*
M2		PCD-CT Br76 70 keV	**PCD-CT Br76 150 keV**	11	0.85	0.75 – 0.93	0.001	*

M2	PCD-CT Br76 110 keV	PCD-CT Br76 150 keV	11	0.47	0.34 – 0.61	0.674	
M3	**EID-CT Br59 iMAR**	PCD-CT Br56 iMAR	11	0.14	0.03 – 0.29	0.002	*
M3	EID-CT Br59 iMAR	PCD-CT Br76 110 keV	11	0.49	0.26 – 0.71	0.933	
M3	EID-CT Br59 iMAR	PCD-CT Br76 150 keV	11	0.42	0.17 – 0.69	0.336	
M3	**EID-CT Br59 iMAR**	PCD-CT Br80	11	0.25	0.08 – 0.43	0.014	*
M3	EID-CT Br64	PCD-CT Br76 110 keV	11	0.40	0.25 – 0.58	0.197	
M3	**EID-CT Br64**	PCD-CT Br80	11	0.18	0.05 – 0.32	0.004	*
M3	PCD-CT Br56 iMAR	PCD-CT Br80	11	0.63	0.47 – 0.77	0.107	
M3	PCD-CT Br76 70 keV	**PCD-CT Br76 110 keV**	11	0.81	0.65 – 0.92	0.003	*
M3	PCD-CT Br76 70 keV	**PCD-CT Br76 150 keV**	11	0.77	0.59 – 0.90	0.006	*
M3	PCD-CT Br76 110 keV	PCD-CT Br76 150 keV	11	0.43	0.28 – 0.57	0.313	

Values of AUC <0.5 indicate higher scores for the "Reference" image. AUC >0.5 indicate higher scores for the "Test" image. Significant differences are marked with *. and the image with the highest score is in bold.

VGC Visual Grading Characteristics. AUC Area under the curve. M1 Metal artifact streaks at the level of several screws. M2 Metal artifact streaks at the level of the distal screw. M3 Metal-bone interface

Table S5 Results of the VGC analysis for image criteria concerning bone.

Image criteria	Reference image	Test image	AUC	95% CI	p-value	Sig.
B1	EID-CT Br59 iMAR	PCD-CT Br56 iMAR	0.53	0.33 – 0.73	0.565	
B1	EID-CT Br59 iMAR	**PCD-CT Br80**	0.79	0.60 – 0.93	0.005	*
B1	EID-CT Br64	**PCD-CT Br80**	0.80	0.63 – 0.94	0.004	*
B1	PCD-CT Br56 iMAR	**PCD-CT Br80**	0.77	0.52 – 0.94	0.007	*
B1	**PCD-CT Br76 70 keV**	PCD-CT Br76 110 keV	0.28	0.18 – 0.38	0.001	*
B1	**PCD-CT Br76 70 keV**	PCD-CT Br76 150 keV	0.20	0.11 – 0.28	0.000	*
B1	**PCD-CT Br76 110 keV**	PCD-CT Br76 150 keV	0.38	0.27 – 0.51	0.044	*
B1	PCD-CT Br76 70 keV	**PCD-CT Br80**	0.66	0.55 – 0.77	0.007	*
B2	EID-CT Br59 iMAR	PCD-CT Br56 iMAR	0.59	0.42 – 0.75	0.183	
B2	EID-CT Br59 iMAR	**PCD-CT Br80**	0.92	0.80 – 0.99	0.001	*
B2	EID-CT Br64	**PCD-CT Br80**	0.91	0.81 – 0.98	0.000	*
B2	PCD-CT Br56 iMAR	**PCD-CT Br80**	0.84	0.68 – 0.95	0.001	*
B2	**PCD-CT Br76 70 keV**	PCD-CT Br76 110 keV	0.22	0.12 – 0.32	0.001	*
B2	**PCD-CT Br76 70 keV**	PCD-CT Br76 150 keV	0.13	0.03 – 0.25	0.000	*
B2	**PCD-CT Br76 110 keV**	PCD-CT Br76 150 keV	0.37	0.25 – 0.48	0.027	*
B2	PCD-CT Br76 70 keV	**PCD-CT Br80**	0.73	0.62 – 0.83	0.002	*
B3	EID-CT Br59 iMAR	PCD-CT Br56 iMAR	0.53	0.35 – 0.70	0.631	
B3	EID-CT Br59 iMAR	**PCD-CT Br80**	0.70	0.48 – 0.90	0.016	*
B3	EID-CT Br64	**PCD-CT Br80**	0.79	0.61 – 0.93	0.004	*
B3	PCD-CT Br56 iMAR	**PCD-CT Br80**	0.71	0.52 – 0.88	0.010	*

B3	**PCD-CT Br76 70 keV**	PCD-CT Br76 110 keV	0.32	0.18 – 0.45	0.009	*
B3	**PCD-CT Br76 70 keV**	PCD-CT Br76 150 keV	0.21	0.09 – 0.33	0.002	*
B3	**PCD-CT Br76 110 keV**	PCD-CT Br76 150 keV	0.39	0.28 – 0.50	0.043	*
B3	PCD-CT Br76 70 keV	**PCD-CT Br80**	0.76	0.64 – 0.85	0.004	*
O1	EID-CT Br59 iMAR	PCD-CT Br56 iMAR	0.56	0.40 – 0.71	0.360	
O1	EID-CT Br59 iMAR	**PCD-CT Br80**	0.72	0.50 – 0.92	0.018	*
O1	EID-CT Br64	**PCD-CT Br80**	0.76	0.57 – 0.91	0.003	*
O1	PCD-CT Br56 iMAR	**PCD-CT Br80**	0.67	0.44 – 0.92	0.023	*
O1	**PCD-CT Br76 70 keV**	PCD-CT Br76 110 keV	0.27	0.15 – 0.39	0.007	*
O1	**PCD-CT Br76 70 keV**	PCD-CT Br76 150 keV	0.16	0.06 – 0.28	0.001	*
O1	**PCD-CT Br76 110 keV**	PCD-CT Br76 150 keV	0.35	0.23 – 0.46	0.023	*
O1	PCD-CT Br76 70 keV	**PCD-CT Br80**	0.68	0.49 – 0.86	0.045	*

Values of AUC <0.5 indicate higher scores for the "Reference" image, AUC >0.5 indicate higher scores for the "Test" image. Significant differences are marked with *, and the image with the highest score is in **bold**.
VGC Visual Grading Characteristics*, AUC* Area under the curve*, B1 Cortical bone, B2 Trabecular architecture, B3 Fracture, O1 Overall bone*

Table S6 Results of the VGC analysis for image criteria concerning soft tissue for each compared pair of image type.

Image criteria	Reference image	Test image	AUC	95% CI	p-value	Sig.
S1	**EID-CT Br40 iMAR**	PCD-CT Br44 iMAR	0.38	0.24 – 0.53	0.049	*
S1	**EID-CT Br40 iMAR**	PCD-CT Br56 iMAR	0.29	0.19 – 0.40	0.006	*
S1	PCD-CT Br44 iMAR	PCD-CT Br44 110 keV	0.51	0.40 – 0.65	0.741	
S1	PCD-CT Br44 iMAR	PCD-CT Br56 iMAR	0.41	0.26 – 0.54	0.101	
S2	EID-CT Br40 iMAR	PCD-CT Br44 iMAR	0.59	0.46 – 0.73	0.125	
S2	EID-CT Br40 iMAR	PCD-CT Br56 iMAR	0.57	0.43 – 0.71	0.303	
S2	**PCD-CT Br44 iMAR**	PCD-CT Br44 110 keV	0.37	0.22 – 0.53	0.038	*
S2	PCD-CT Br44 iMAR	PCD-CT Br56 iMAR	0.46	0.30 – 0.62	0.477	
O2	EID-CT Br40 iMAR	PCD-CT Br44 iMAR	0.50	0.31 – 0.67	0.948	
O2	EID-CT Br40 iMAR	PCD-CT Br56 iMAR	0.45	0.31 – 0.63	0.521	
O2	**PCD-CT Br44 iMAR**	PCD-CT Br44 110 keV	0.31	0.18 – 0.48	0.024	*
O2	PCD-CT Br44 iMAR	PCD-CT Br56 iMAR	0.45	0.28 – 0.65	0.330	

Values of AUC <0.5 indicate higher scores for the "Reference" image, AUC >0.5 indicate higher scores for the "Test" image. Significant differences are marked with *, and the image with the highest score is in **bold.**
VGC Visual Grading Characteristics*, AUC* Area under the curve*, S1 Anterior cruciate ligament, S2 Sartorius muscle, O2 Overall soft tissue*

Fig. S1 Representative example of VOI placement.

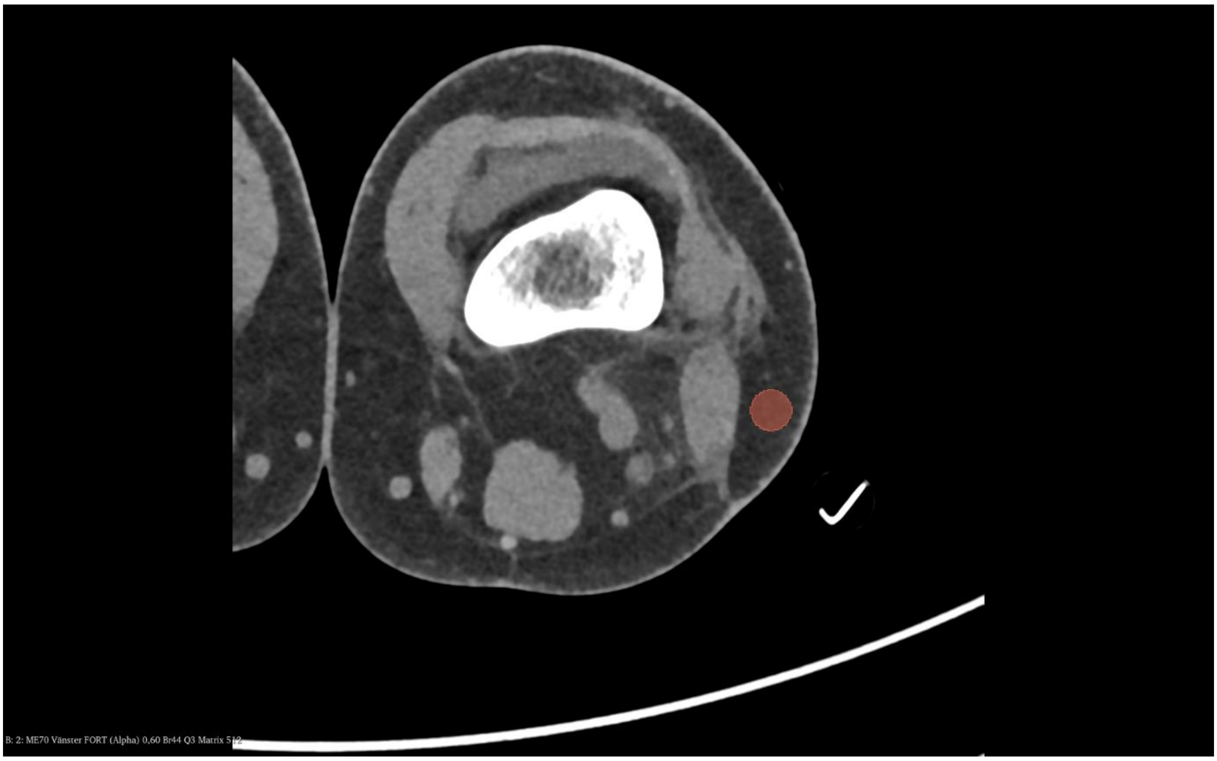

Fig. S2 Representative axial CT images of smooth-kernel reconstructions at the level of the distal femur.

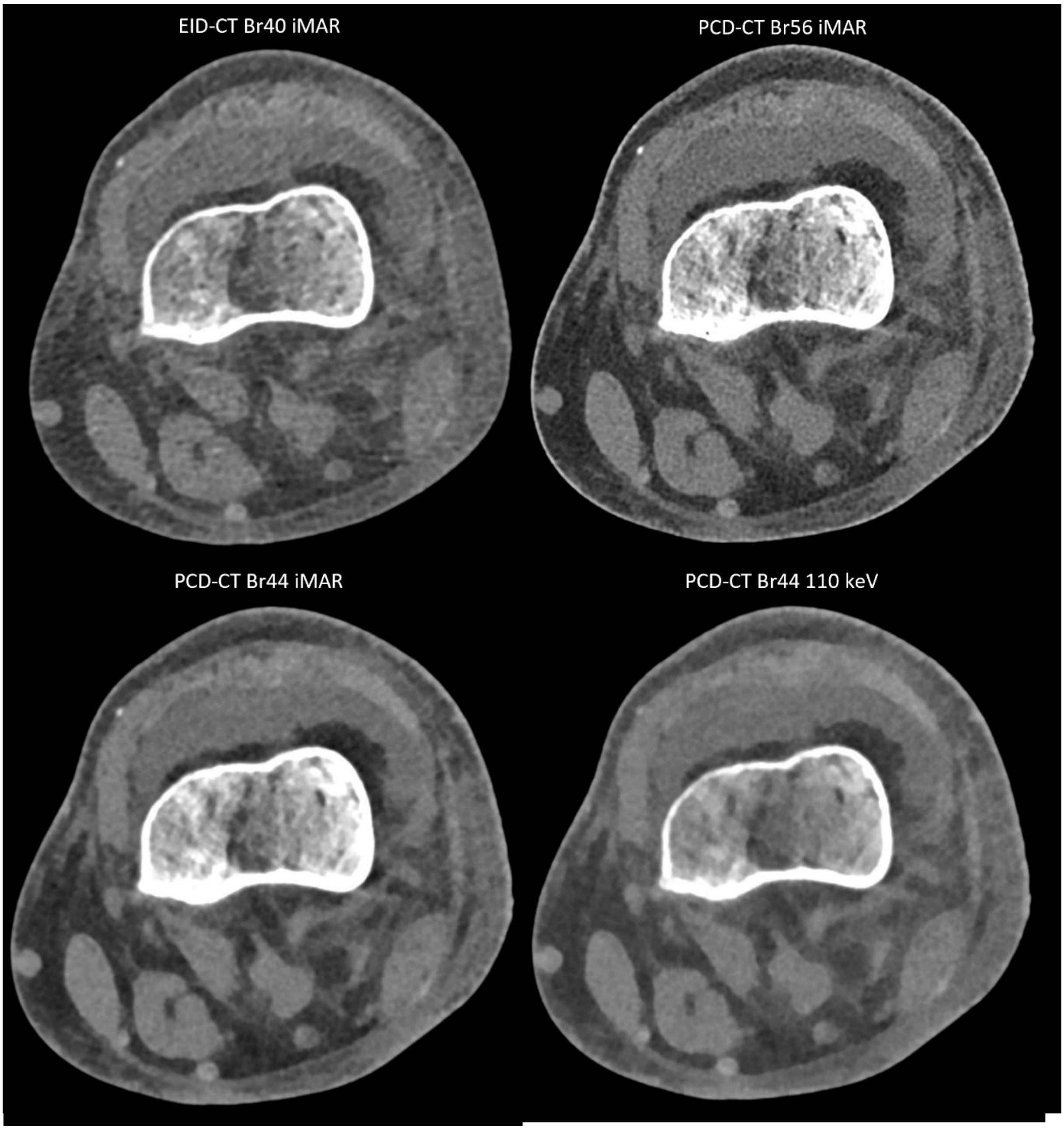

Supplement: Supplementary file 1 — Additional File 1: Table S1. Image evaluation criteria and interpretation of Likert scores in the second part of the evaluation. Table S2. Frequency table of scores from the first part of the image quality evaluation. Table S3. Frequencies and percentages of observer scores for the side-by-side comparison of image reconstruction types compared to the corresponding reference reconstruction (no iMAR, 70 keV). Table S4. Results of the VGC analysis for image criteria concerning metal artifacts. Table S5. Results of the VGC analysis for image criteria concerning bone. Table S6. Results of the VGC analysis for image criteria concerning soft tissue for each compared pair of image types. Fig. S1. Representative example of VOI placement. Fig. S2. Representative axial CT images of smooth-kernel reconstructions at the level of the distal femur. [file 41747_2026_761_MOESM1_ESM.pdf]
